# The First Known Documented Case of Ewingella Americana Urinary Tract Infection

**DOI:** 10.7759/cureus.35640

**Published:** 2023-03-01

**Authors:** Jason Hourizadeh, Justin Joy, Joseph I Berger, Hanady Zainah

**Affiliations:** 1 Internal Medicine, St. John's Riverside Hospital, Yonkers, USA

**Keywords:** hemodialysis, immunocompromised, urinary tract infection, multidrug-resistant, ewingella americana

## Abstract

We present a 73-year-old male with a history of end-stage renal disease (ESRD) on dialysis, type 2 diabetes mellitus, coronary artery disease status post stents, prostate carcinoma status post radiation, and prostatectomy, with recurrent bladder neck contracture requiring suprapubic catheter, left urethral stricture with nephrostomy tube placement, penile implant, and recurrent urinary tract infections, who presented to the emergency room complaining of constant bilateral groin pain for one day. Physical exam was significant for suprapubic tenderness and a chronic suprapubic catheter and left-sided nephrostomy tube. An initial examination of the patient’s urine revealed turbid, yellow-colored fluid, positive for white blood cells, leukocyte esterase, and bacteria. A urine culture was obtained, which returned positive for *E. americana, *with >100,000colony-forming units (CFUs)as well as *Enterococcus faecalis (E. faecalis) *demonstrating low colony counts. The patient was treated with a seven-day course of meropenem 1 gm twice daily, which improved of his symptoms, and then completed a 10-day course of ertapenem 500 mg daily. The patient received a five-day course of vancomycin 1 gm on dialysis days for additional coverage of *E. faecalis*, despite low colony counts. This is the first documented case of a urinary tract infection caused by *E. americana*. The organism is primarily found in immunocompromised individuals, and a debate is still ongoing as to whether it is a true pathogen or exists primarily as an opportunistic infection. We suggest further inquiry and study of this resistant organism are paramount in establishing its role in both immunocompromised as well as immunocompetent individuals. *E. americana* is a multidrug-resistant organism, which to date has sparse documentation regarding its prevalence and potential for morbidity, especially in compromised individuals. In the era of increasing antibiotic resistance, we suggest that more research is needed to understand the pathogenicity of *E. americana*.

## Introduction

*Ewingella americana* (*E. americana)* is a gram-negative, lactose-fermenting, oxidase-negative, catalase-positive, indole-negative, facultative anaerobic bacillus in the *Enterobacteriaceae *family first described in 1983 by Grimont et al., named after the American bacteriologist William H. Ewing [1]. *Ewingella* was originally thought to be related to the genus *Cedecea *and the family *Enterobacteriaceae*, but now, it has a new genus and species, named *E. americana* [2]. The biochemical differences found in what was previously called Enteric Group 40, including a negative malonate test, negative production of lipase, and negative production of gas from glucose, led to the creation of this new genus and species [2]. As of this current case report, it is the only species within its genus [3]. The bacteria grow on 5% sheep blood agar aerobically and anaerobically as well as on MacConkey agar incubated overnight at 37°C [4]. Most of the *E. americana* strains are methyl red-positive, Voges-Proskauer-positive, Simmons citrate-positive, lysine decarboxylase-negative, ornithine decarboxylase-negative, and arginine dihydrolase-negative [4]. However, it is known that it can survive in water and grow at 4°C [5]. Ryoo et al. stated that a potential source of *E. americana* infection could be the contamination of the catheter or improper hand hygiene [5]. Other reports state that *E. americana* can be found in citrate solutions, ice baths, running water, and some vegetables [4].

*E. americana* is primarily seen in immunocompromised patients, such as patients with diabetes mellitus, end-stage renal disease (ESRD), those who have undergone chemotherapy or bone marrow transplantation, and those who use mercaptopurine and is thus considered an opportunistic pathogen [1]. However, when *E. americana* infects the healthy population, it is a mild infection and presents as conjunctivitis and/or a respiratory infection with a rapid resolution, and the need for antibiotic treatment is not fully described [6]. A review of the literature demonstrated *E. americana*-associated bacteremia, pneumonia, peritonitis, conjunctivitis, meningitis [4], osteomyelitis, and septic arthritis, with the most common site of isolation being the blood [1,7]. It is important to mention that Farmer et al. reported one case of *E. americana* isolated from the urine; however, no documentation of a distinct infection was noted [8].

We report the first documented case of a urinary tract infection caused by *E. americana*.

## Case presentation

We present a case of a 73-year-old male with a past medical history of ESRD on dialysis, type 2 diabetes mellitus (last known HbA1c 6.5%), coronary artery disease status post stents, prostate carcinoma status post radiation and prostatectomy, with recurrent bladder neck contracture requiring suprapubic catheter, left urethral stricture with nephrostomy tube placement, penile implant, and recurrent urinary tract infections not on prophylactic antibiotics, who presented to the emergency room complaining of constant bilateral groin pain for one day.

Vitals on admission were notable for tachycardia and mild hypoxia on room air (Table 1).

**Table 1:** Vital signs on admission F: Fahrenheit; Bpm: Beats per minute.

Vital signs on admission	
Temperature	98.6°F
Pulse rate	95 bpm
Blood pressure	139/63
O_2_ saturation	93% on room air

Physical exam was significant for suprapubic tenderness, a chronic suprapubic catheter, and a left-sided nephrostomy tube. An initial examination of the patient’s urine revealed turbid, yellow-colored fluid, positive for white blood cells, leukocyte esterase, and bacteria (Table 2).

**Table 2:** Urinalysis WBC/μl: White blood cells per microliter; RBC/μl: Red blood cells per microliter; Bacteria/μl: Bacteria/microliter.

Urinalysis (Foley)	
Color	Yellow
Appearance	Turbid
Urine pH	8.5
Urine protein	3+
Urine glucose	Negative
Urine ketone	Negative
Urine blood	2+
Urine nitrite	Negative
Urine bilirubin	Negative
Urine urobilinogen	0.2
Urine leukocyte esterase	3+
Urine WBC (WBC/μl)	17,877
Urine RBC (RBC/μl)	671.3
Urine casts (casts/μl)	13
Urine bacteria (bacteria/μl)	>9,000

A urine culture was obtained, which returned positive for *E. americana* with >100,000 colony-forming units (CFUs) resistant to cephalosporins, ampicillin, aztreonam, and trimethoprim/sulfamethoxazole as well as *Enterococcus faecalis* (*E. faecalis*) demonstrating low colony counts (Tables 3, 4).

**Table 3:** Urine culture CFUs: Colony-forming units.

Urine culture (Foley)		
Organism	Ewingella americana	Enterococcus faecalis
CFUs	100,000 CFUs	40,000-50,000 CFUs

**Table 4:** Urine culture sensitivities/MIC MIC: Minimum inhibitory concentration.

Urine culture sensitivities/minimum inhibitory concentration		
Antibiotic	Ewingella americana	Enterococcus faecalis
Amikacin	<16	-
Ampicillin/Sulbactam	>16	<2
Aztreonam	16/8	-
Cefazolin	>16	-
Cefotaxime	>32	-
Ceftazidime	16	-
Ceftriaxone	>32	-
Cefuroxime	>32	-
Daptomycin	-	1
Ertapenem	<1	-
Gentamicin	<4	-
Imipenem	<1	-
Levofloxacin	<2	2
Linezolid	-	2
Meropenem	<1	-
Nitrofurantoin	64	<32
Penicillin	-	2
Piperacillin/Tazobactam	<16	-
Tetracycline	-	>8
Tigecycline/Tygacil	<2	-
Tobramycin	<4	-
Trimethoprim/Sulfamethoxazole	>2/38	-
Vancomycin	-	2

Based on recommendations of the Clinical and Laboratory Standards Institute, comprehensive antimicrobial testing was done using *E. coli* as a control to detect antibiotic resistance in *Ewingella* (Table 5) [9].** **The penile culture was positive for extended-spectrum beta-lactamase (ESBL) producing *Klebsiella Pneumonia* and *E. faecalis* (Tables 5, 6).** **Since the number of colonies was insignificant, they were not considered causes of the patient's urinary tract infection (UTI) and therefore did not warrant treatment. Blood cultures were negative. 

**Table 5:** Ewingella antibiotic susceptibilities μg: Microgram; mm: Millimeter.

Evaluation standards for *Ewingella* antibiotic sensitivity				
Antibiotic	Dose (μg)	Resistance (mm)	Intermediate (mm)	Sensitive (mm)
Ampicillin	10	≦13	14-16	≧17
Aztreonam	30	≦15	16-18	≧19
Cefazolin	30	≦14	15-17	≧18
Cefixime	5	≦15	16-18	≧19
Ceftriaxone	30	≦13	14-20	≧21
Clindamycin	22	≦14	15-20	≧21
Ciprofloxacin	5	≦15	16-20	≧21
Erythrocin	15	≦13	14-22	≧23
Gentamicin	10	≦12	13-14	≧15
Kanamycin	30	≦13	14-17	≧18
Novobiocin	5	≦12	13-16	≧17
Ofloxacin	5	≦12	13-15	≧16
Rifampicin	5	≦16	17-19	≧20
Streptomycin	10	≦11	12-14	≧15
Tetracycline	30	≦14	15-18	≧19
Vancomycin	30	≦14	15-16	≧17

**Table 6:** Urine culture sensitivities/MIC MIC: Minimum inhibitory concentration; ESBL: Extended-spectrum beta-lactamase.

Penile culture sensitivities/minimum inhibitory concentration		
Antibiotic	Klebsiella Pneumonia - ESBL	Enterococcus faecalis
Amikacin	<16	-
Ampicillin	-	<2
Ampicillin/Sulbactam	16/8	-
Cefepime	>16	-
Daptomycin	-	1
Ertapenem	<1	-
Erythromycin	-	>4
Gentamicin	<4	-
Imipenem	<1	-
Levofloxacin	<2	2
Linezolid	-	2
Meropenem	< 1	-
Penicillin	-	2
Piperacillin/Tazobactam	<16	-
Tetracycline	-	>8
Tigecycline/Tygacil	<2	-
Tobramycin	<4	-
Trimethoprim/Sulfamethoxazole	>2/38	-
Vancomycin	-	1

On admission, the** **patient's suprapubic catheter was exchanged, and he underwent a CT scan of the abdomen and pelvis, which did not show hydronephrosis or perinephric stranding.** **The patient was treated with a seven-day course of meropenem 1 gm twice daily, which showed marked improvement in his symptoms, and then completed a 10-day course of ertapenem 500 mg daily. The patient also received a five-day course of vancomycin 1 gm on dialysis days for additional coverage of *E. faecalis*, despite low colony counts [10,11] (Tables 6, 7). Unfortunately, the patient did not return to the hospital or the hospital clinic following treatment and was lost to follow-up.

**Table 7:** Penile culture ESBL: Extended-spectrum beta-lactamase.

Penile culture		
Organism	Klebsiella Pneumonia - ESBL	Enterococcus faecalis
Number of colonies	Few seen	Rare

## Discussion

We present the first documented case of a urinary tract infection caused by *E. americana*. *E. americana* has been seen in a variety of clinical samples such as sputum, blood, conjunctiva, and wounds [6,7]. Studies have suggested that patients infected by this organism have underlying comorbidities and immunocompromising conditions including complicated surgeries, drug abuse, renal failure, diabetes, prolonged hospitalizations, indwelling catheters, and immunosuppressive therapy. While clinical infection with *E. americana* has been documented outside of the urinary tract, this is the first known case of a symptomatic urinary tract infection caused by *E. americana *[1,4-7,10]. Our patient’s extensive past medical history is congruent with the patient population previously seen infected with this organism.

Ryoo et al. speculated that the source of the infection can be the contamination of a catheter or improper hand hygiene [1,5-7] The natural habitat of *E. americana* is unknown; however, some reports suggest that catheters and running waters can harbor the organism [4]. Khurana et al. concluded that patients with indwelling peritoneal catheters can be infected due to improper sterile technique and poor hand hygiene [7]. This puts our patient at risk for infection from *E. americana* because he has multiple risk factors that increase his risk such as being a diabetic, having a history of cancer, and having multiple catheters placed into his urinary tract. Therefore, one may postulate that our patient acquired this infection via his chronic catheter use or improper hygiene while handling the said catheters.

In general, *E. americana* has been shown to be resistant to first- and second-generation cephalosporins, although sensitive to third- and fourth-generation cephalosporins, with variable sensitivities to penicillin; however, multidrug-resistant organisms have been documented [6]. In a study conducted by Stock et al., the susceptibility of 20* E. americana *strains* *to 72 different antibiotics was tested. They concluded that *Ewingella* was naturally resistant or of intermediate susceptibility to narrow-spectrum cephalosporins, specifically cefaclor, loracarbef, cefazolin, cefuroximine, cefoxitin, and fosfomycin [12].* E. americana* is also resistant to antibiotics for which most *Enterobacteriaceae *are naturally resistant such as benzylpenicillin, oxacillin, erythromycin, roxithromycin, clarithromycin, lincomycin and clindamycin, dalfopristin-quinupristin, telithromycin and ABT-773, linezolid, teicoplanin and vancomycin, fusidic acid and rifampicin, and nitrofurantoin [12].

In at-risk patients, including but not limited to immunocompromised patients, prompt antibiotic therapy is mandatory. Our case is notable not only for the unique organism but for its incredibly multidrug-resistant nature (Table 4). Also, based on the recommendations of the Clinical and Laboratory Standards Institute, they concluded that *E. americana* is resistant to many antibiotics (Table 5) [9]. It was found that *E. americana *contains about 67 putative virulence genes [9]. The virulence features include the secretion system, adherence, invasion, chemotaxis, motility, immune evasion, and pore-forming toxins [9]. These features can be the reason for the multidrug-resistant nature of *E. americana.*

While *E. americana* is primarily found in immunocompromised individuals, as of date, a debate is still ongoing as to whether it is a true pathogen or exists primarily as an opportunistic infection [1,6]. We suggest that further inquiry and study of this organism are required in establishing its role in both immunocompromised as well as immunocompetent individuals.

## Conclusions

This is the first documented case of *E. americana* causing a urinary tract infection. *E. americana* is a rarely seen, often opportunistic infection, that is currently the only species within its genus. Due to its documented history of multidrug resistance, we believe that further research on its pathogenesis and its effect on morbidity and mortality needs to be conducted, especially in immunocompromised and high-risk individuals.
